# Determinants of day–night difference in blood pressure, a comparison with determinants of daytime and night-time blood pressure

**DOI:** 10.1038/jhh.2016.14

**Published:** 2016-03-17

**Authors:** M D Musameh, C P Nelson, J Gracey, M Tobin, M Tomaszewski, N J Samani

**Affiliations:** 1 Department of Cardiovascular Sciences, University of Leicester, British Heart Foundation Cardiovascular Research Centre, Glenfield Hospital, Leicester, UK; 2 National Institute for Health Research Leicester Cardiovascular Biomedical Research Unit, Glenfield Hospital, Leicester, UK; 3 Department of Health Sciences, University of Leicester, University Road, Leicester, UK

## Abstract

Blunted day–night difference in blood pressure (BP) is an independent cardiovascular risk factor, although there is limited information on determinants of diurnal variation in BP. We investigated determinants of day–night difference in systolic (SBP) and diastolic (DBP) BP and how these compared with determinants of daytime and night-time SBP and DBP. We analysed the association of mean daytime, mean night-time and mean day–night difference (defined as (mean daytime−mean night-time)/mean daytime) in SBP and DBP with clinical, lifestyle and biochemical parameters from 1562 adult individuals (mean age 38.6) from 509 nuclear families recruited in the GRAPHIC Study. We estimated the heritability of the various BP phenotypes. In multivariate analysis, there were significant associations of age, sex, markers of adiposity (body mass index and waist–hip ratio), plasma lipids (total and low-density lipoprotein cholesterol and triglycerides), serum uric acid, alcohol intake and current smoking status on daytime or night-time SBP and/or DBP. Of these, only age (*P*=4.7 × 10^−5^), total cholesterol (*P*=0.002), plasma triglycerides (*P*=0.006) and current smoking (*P*=3.8 × 10^−9^) associated with day–night difference in SBP, and age (*P*=0.001), plasma triglyceride (*P*=2.2 × 10^−5^) and current smoking (3.8 × 10^−4^) associated with day–night difference in DBP. 24-h, daytime and night-time SBP and DBP showed substantial heritability (ranging from 18–43%). In contrast day–night difference in SBP showed a lower heritability (13%) while heritability of day–night difference in DBP was not significant. These data suggest that specific clinical, lifestyle and biochemical factors contribute to inter-individual variation in daytime, night-time and day–night differences in SBP and DBP. Variation in day–night differences in BP is largely non-genetic.

## Introduction

Night-time blood pressure (BP) is typically lower than BP during the day. Non-dipping of nocturnal BP and elevated nocturnal BP (nocturnal hypertension) have both emerged as independent risk factors for cardiovascular disease as well as cardiovascular and all-cause mortality.^1, 2, 3, 4, 5, 6, 7, 8^ Although the prognostic importance of non-dipping has been confirmed in multiple studies, especially since the advent of 24 h non-invasive ambulatory BP (ABP) monitoring, less information exists on determinants of diurnal variation in BP and whether these are similar to or differ from determinants of daytime and night-time BP.

In this study, we have taken advantage of data, including ABPs, collected in the Genetic Regulation of Arterial Pressure in Humans in the Community (GRAPHIC) Study^9, 10^ to identify phenotypic characteristics associated with day–night difference in systolic (SBP) and diastolic (DBP) BPs and compare these with characteristics associated with mean daytime and nocturnal SBP and DBP in the same individuals. Because the GRAPHIC Study has a nuclear family design,^9, 10^ we were also able to assess the extent of heritability of different time-related BP phenotypes in the same subjects and in particular assess the heritability of night–day differences in BP that has been studied less extensively.

## Materials and methods

### Study population and phenotyping

Details of recruitment and phenotyping of the GRAPHIC subjects are described elsewhere.^9, 10^ The study was approved by the Leicestershire Research Ethics Committee (Ref No. 6463) and all subjects provided written informed consent. Recruitment took place between 2002 and 2005. Briefly, nuclear families (all of white European ancestry) with both parents (aged 40–60 years) and two adult (⩾18 years) offspring were identified through general practices in Leicestershire, UK, and invited to participate through an invitation letter sent through the mother. No selection criteria were applied, except for exclusion of families known to have a member with advanced renal disease.

Participants had a detailed history taken using a questionnaire and were examined by research nurses following standard protocol, to include measurements of height, weight and waist–hip ratio. Three clinic BP readings were made using an Omron HEM-705CP digital BP monitor with an appropriate size cuff (on the non-dominant arm with an interval of at least 3 min between readings). Clinic BP was defined as the mean of the second and third readings. The first BP reading was discarded to reduce any impact of the alerting response. Blood samples were obtained for biochemical and DNA analysis and participants collected 24-h urine samples. Plasma and urinary electrolytes and creatinine, plasma lipids (non-fasting) and C-reactive protein (CRP) were measured using standard assays in certified clinical biochemistry laboratories. Estimated glomerular filtration rate was calculated using the CKD-EPI (Chronic Kidney Disease Epidemiology Collaboration) equation.^11^

### ABP measurements, exclusion of subjects and definition of day–night difference in BP

ABP was measured using a Spacelabs 90207 monitor (Spacelabs, Wokingham, UK) for 26 h. The first 2 h of each record was discarded to avoid an alerting response.^9^ ABP measurements were done every 30 min during the daytime and hourly at night. Of the 2024 individuals from 520 nuclear families recruited in GRAPHIC, individuals who were receiving anti-hypertensive medications (*n*=135) were excluded from the current analysis to avoid any confounding caused by modulation of diurnal variation in BP by drugs. Individuals who reported being shift workers (*n*=86) were also excluded. Finally, subjects (*n*=241) who did not achieve >75% of BP readings during either daytime, night-time or both were also excluded to avoid any bias due to poor quality assessment of day–night differences in BP, leaving a total of 1562 subjects (from 509 families) in the present analysis. There were 188 families with 4 members, 203 families with 3 members, 82 families with 2 members and 35 families with 1 member.

Narrow periods were used to define daytime (1000 –2000 hours) and night-time (0000–0600 hours) as previously described.^2, 5^ Day–night difference in BP was defined as a percentage decline using the formula:


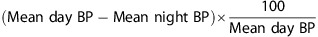


This was done separately for SBP and DBP. Ratio of average night-time over average daytime SBP and DBP, another measure of day–night difference in BP,^2, 12^ was also calculated and its correlation with percentage decline in SBP and DBP was examined.

### Statistical analysis

Univariate and multivariate stepwise regression, using generalised estimating equations, accounting for the family structure, were adopted to assess the associations between daytime, night-time and day–night differences in SBP and DBP with demographic, lifestyle, BP and biochemical parameters as described. All analyses were carried out using the statistical software package STATA v13.1 (StataCorp, College Station, TX, USA).

Heritability of daytime, night-time, day–night difference as well as 24-h SBP and DBP was calculated using variance components in the software package Sequential Oligogenic Linkage Analysis Routines (SOLAR, Texas Biomedical Research Institute, San Antonio, TX, USA).^13^ Adjustments for age, age^2^ (because of the two generational structure of GRAPHIC) and sex were made when calculating heritability. In addition for night–day differences in SBP, the model was adjusted for the respective mean day BP.

## Results

**Figure 1:**
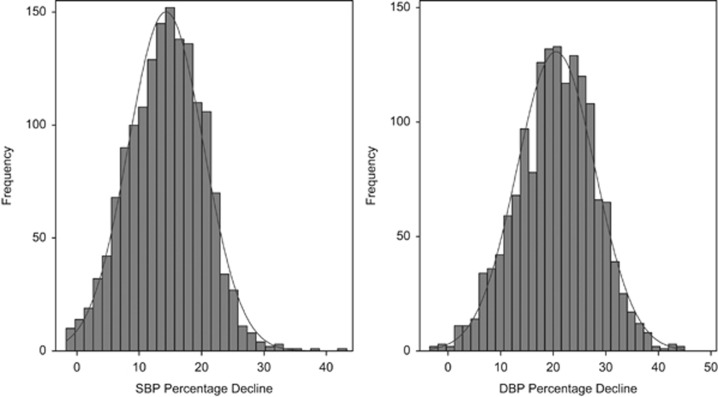
Distribution of day–night difference in systolic (SBP) and diastolic blood pressures (DBP). Histograms showing the distribution of day–night difference in SBP (left panel) and DBP (right panel) defined as percentage decline over respective daytime BP.

The characteristics of the subjects analysed partitioned by generation and sex are shown in Table 1. We have previously shown that subjects recruited in the GRAPHIC Study are representative of the general UK-European ancestry population.^9^ The distributions of day–night difference in SBP and DBP plotted as a percentage decline from respective daytime BPs are shown in Figure 1. Both show near-normal Gaussian patterns with a range from virtually minimal decline to an over 40% decline in BP. The distributions in night–day difference in SBP and DBP calculated as a ratio of average night-time BP over average daytime BP are shown in Supplementary Figure 1. The patterns are very similar to that for percentage declines in BP and the two measures are perfectly correlated (Supplementary Figure 2) indicating that they represent the same information. There was also a correlation between the day–night difference in SBP and DBP (Supplementary Figure 3).

The results of the univariate association analysis of day–night differences in SBP and DBP with clinical and biochemical characteristics are shown in Supplementary Tables 1 and 2, respectively, together with the corresponding analyses for daytime and night-time mean BPs. All associations that had a *P*-value of <0.1 for any of the SBP or DBP phenotypes in the respective univariate analysis were taken forward into the multivariate stepwise regression analyses for each. The results of these analyses are summarised in Table 2 for SBP phenotypes and Table 3 for DBP phenotypes.

For SBP, several clinical and biochemical factors showed independent associations with either daytime, night-time or both BPs (Table 2). Notably, there was a positive correlation of mean daytime SBP with age, body mass index (BMI) and alcohol intake while, as anticipated, on average women had a lower daytime SBP of almost 5 mm Hg compared with men. Among the biochemical factors there was a positive correlation of daytime SBP with plasma triglyceride level, urate level and CRP level. For nocturnal SBP more variables showed significant associations (Table 2). Notably, although the associations with sex, BMI, alcohol intake, plasma triglyceride and CRP were in the same direction as those for daytime SBP, the association of nocturnal SBP with age was inversed and there was a substantial effect of smoking status on nocturnal SBP with current smokers having a significantly lower nocturnal SBP (~3 mm Hg). As a consequence of these variable effects, the two factors with the greatest impact on day–night difference in SBP were age and smoking status (Table 2). Total plasma cholesterol also had a significant positive correlation with day–night difference in SBP due to a significant negative association with nocturnal SBP (Table 2).

For DBP, age was not associated with day–night difference in BP as it had positive correlations of a similar degree with both daytime and night-time (DBP; Table 3). However, smoking status showed the same difference as seen with SBP, again due to a strong inverse association with nocturnal DBP (Table 3). Both plasma cholesterol level and triglyceride level showed significant positive correlations with day–night difference in DBP due to differential (although same direction) associations with daytime and nocturnal DBP (Table 3). BMI was not associated with any DBP phenotypes but waist–hip ratio showed positive associations with both average daytime and night-time DBP but not with day–night difference (Table 3).

In a sensitivity analysis, we examined whether the associations observed with day–night difference in SBP and DBP were influenced by mean day BPs, by additionally adjusting for these variables. As shown in Tables 2 and 3, most of the associations remained significant except the association of night–day difference in DBP with cholesterol became non-significant while that with age became significant (Table 3).

Estimates of heritability of 24 -h, daytime, night-time and day–night difference for SBP and DBP are shown in Table 4. 24-h, daytime and night-time SBP and DBP all showed substantial heritability with DBP phenotypes on the whole showing higher levels than SBP phenotypes. In contrast, day–night difference in DBP did not show significant heritability and heritability of day–night difference in SBP was lower than for other SBP phenotypes.

## Discussion

Several studies have demonstrated that nocturnal hypertension and a reduced difference in day–night BP (non-dipping) are important prognostic markers of cardiovascular risk.^1, 2, 3, 4, 5, 6, 7, 8^ Although some studies have used categorical definitions for non-dipping, day–night difference in SBP and DBP, as we show here, defined either as percentage decline (over daytime BP) or as night–day ratio, are continuous normally distributed phenotypes. Hence, in investigating their determinants, it is appropriate to study them as quantitative rather than categorical traits. Furthermore, because percentage decline in BP and night–day ratio in BP are perfectly correlated (Supplementary Figure 2), the determinants we have identified for percentage decline in SBP and DBP also apply equally for their night–day ratios.

Several previous studies have tried to identify determinants of non-dipping (reviewed by Routledge and McFetridge-Durdle^14^). However, this has largely been done in individuals with hypertension and often in those receiving anti-hypertensive treatment.^14, 15, 16, 17, 18, 19, 20, 21, 22^ Although the findings from such studies are clearly of relevance to understanding the impact of variation in night–day difference in BP on cardiovascular outcomes, generalisability of any determinants identified is less clear. In this context, the GRAPHIC study has the advantage of being population based with detailed clinical and biochemical characterisation. Furthermore, we excluded subjects on anti-hypertensive medication (but not those with elevated BP not receiving medication) from the analysis to avoid any confounding.

In univariate analysis, we observed many factors associated with both SBP and DBP, including age, sex (lower in females), markers of adiposity (BMI and waist–hip ratio), plasma lipids (notably low-density lipoprotein cholesterol and triglycerides), serum uric acid and alcohol intake (Supplementary Tables 1 and 2), although only some of these were independently significant in multivariate analysis (Tables 2 and 3). Interestingly, although 24-h urinary sodium (a potential marker of sodium intake) was associated with SBP and DBP in univariate analysis, neither showed associations in the multivariate analysis and renal function as estimated by estimated glomerular filtration rate was not associated with either SBP or DBP. Among the markers of adiposity, waist–hip ratio was more strongly associated with DBP than BMI (and retained in the multivariate analysis), while BMI was more strongly associated with SBP.

Specific and somewhat distinct factors showed an association with day–night differences in SBP and DBP. For SBP, the most significant factor was age, with a greater night–day difference with increasing age (Table 2). In contrast, there was a negative association between increasing age and night–day difference that became apparent after adjustment for mean day DBP (Table 3). There was a positive association between total cholesterol and day–night difference in SBP and DBP although the association with the latter became non-significant after adjustment for daytime DBP. Plasma triglyceride levels showed an inverse association with night–day differences in both SBP and DBP. There was a borderline significant association of alcohol intake with greater day–night difference in DBP (Table 3), which was not significant after adjustment for daytime DBP. The association between plasma triglyceride level and night–day differences in SBP and DBP have not been reported previously and require further corroboration. The mechanism(s) underlying these associations are also not clear. Our sensitivity analysis suggests that these associations are not due to a differential relationship with daytime versus night-time BP.

Perhaps the most surprising finding was the association of current smoking with greater day–night difference in both SBP and DBP, which remained after adjustment for the respective daytime BPs (Tables 2 and 3). The relationship of smoking with BP is complex with several studies showing an acute BP rise with smoking due at least in part to sympathetic activation.^23, 24^ With regard to the longer-term relationship between smoking and BP, studies have shown higher, lower and no differences in BP between smokers and non-smokers.^23, 25, 26, 27^ Few studies have explored any association between smoking status and day–night differences in BP using ambulatory monitoring, especially in population-based cohorts. Consistent with our findings, Mikklesen *et al.*^28^ observed lower nocturnal SBP and DBP in 352 normotensive Dutch subjects aged 20–79 years, as well as lower daytime DBP but did not report on day–night differences. In a small study of 18 healthy smokers and 18 matched healthy non-smokers, Hansen *et al.*^29^ observed lower nocturnal SBP and DBP values in the smokers, and similar to our findings, no difference in daytime SBP or DBP resulting in a greater day–night difference in both SBP and DBP in smokers. Interestingly, this difference was attenuated in 16 diabetics and 16 matched controls. In a cohort of 2042 white hypertensive subjects who are untreated, Schillaci *et al.*^30^ demonstrated an association between smoking and greater day–night BP drop in females only. The mechanism for the differential impact of smoking on nocturnal SBP and DBP remains unclear but could relate to the immediate attenuation of any sympathetic influences from the temporary cessation of smoking during sleep.^23, 24^

We did not see previously reported associations of several clinical and biochemical phenotypes with BP dipping, for example, diabetes mellitus, obesity, CRP, renal function (estimated glomerular filtration rate) and urinary salt excretion.^14, 15, 31, 32, 33, 34^ A likely explanation is that most of the previous studies have been undertaken in patients with hypertension and/or end organ damage,^3, 15, 34, 35, 36, 37^ where some of the variables (for example, CRP and estimated glomerular filtration rate) may show a broader range. Furthermore, it should be noted that because of the low prevalence of diabetes in GRAPHIC (1%, Table 1), we were only able to examine the association with blood glucose as a quantitative trait and did not see any association (Supplementary Tables 1 and 2).

An area of current interest is the heritability of different BP traits especially with the observation that some genetic loci may only affect specific traits, for example, pulse pressure.^38, 39^ Very few studies have examined heritability of different time-related BP traits. In a study of 240 European-American and 190 African-American twins with a mean age of 17.2±3.4 years, Wang *et al.*^40^ found heritabilities of 0.70 and 0.68 for SBP and 0.70 and 0.64 for DBP for daytime and night-time, respectively. They also observed heritabilities of 0.59 and 0.81 for systolic and diastolic dipping (defined as a more than 10% fall in night-time BP of daytime values).^40^ In another study of 260 healthy siblings aged 38.3±8.6 years from 118 Swedish families, Fava *et al.*^41^ reported a heritability of 29% for nocturnal SBP dipping (defined as the night-to-day ratio) but that for DBP was not significant. Here given the nuclear family design of the GRAPHIC Study and the availability of ABP measurements, we were able to undertake the largest analysis to date of heritability of different BP phenotypes in the same subjects. We confirmed significant heritabilities for 24 h as well as daytime and night-time SBP and DBP with the estimates for DBP somewhat higher than those for SBP. In contrast, the heritabilities for day–night differences in BP were much weaker with that for day–night difference in DBP being non-significant. Our findings for the overall heritabilities of SBP and DBP are similar to that reported in the literature (~30–50%).^42, 43, 44^ The higher values reported by Wang *et al.*^40^ may be related to the twin design of their cohort that can sometimes overestimate the genetic contribution.^45^ Our finding for heritability of night–day SBP difference in SBP is also lower than that reported by Fava *et al.*^41^ The reason for this is unclear although it is perhaps noteworthy that the BP dipping variables in the Fava *et al.* subjects were not normally distributed, suggesting possible selection bias. In any case, our findings suggest that day–night differences in SBP and DBP are largely driven by non-genetic mechanisms.

Although the structure of the GRAPHIC cohort allowed us to make observations on night–day determinants of BP in the general population and enabled examination of its heritability, several limitations of our study need to be highlighted. First, we lacked information on physical activity and on psychosocial factors, including depression and socioeconomic status that have been reported to impact on nocturnal BP dipping,^14^ to include in our analysis. Second, although our analysis is based on participants having at least 75% daytime and night-time successful ABP measurements (equating to at least 14 daytime readings and 5 night-time readings), the frequency of measurements is less than currently recommended in the ESH-ESC guidelines.^46^ Similarly, for clinic BP, to reduce the impact of any alerting response, the first reading was discarded but this is again not currently recommended in the guidelines. Finally, by excluding hypertensive subjects taking medication, we perhaps excluded some individuals with more extreme BPs that may have impacted on the findings.

In summary, by analysing a large population-based cohort of nuclear families with ABP we have identified specific clinical, lifestyle and biochemical factors that contribute to inter-individual variation in daytime, night-time and day–night differences in SBP and DBP. We further show the substantial heritability of both daytime and nocturnal BP but observed that variation in day–night differences in BP is largely non-genetic.


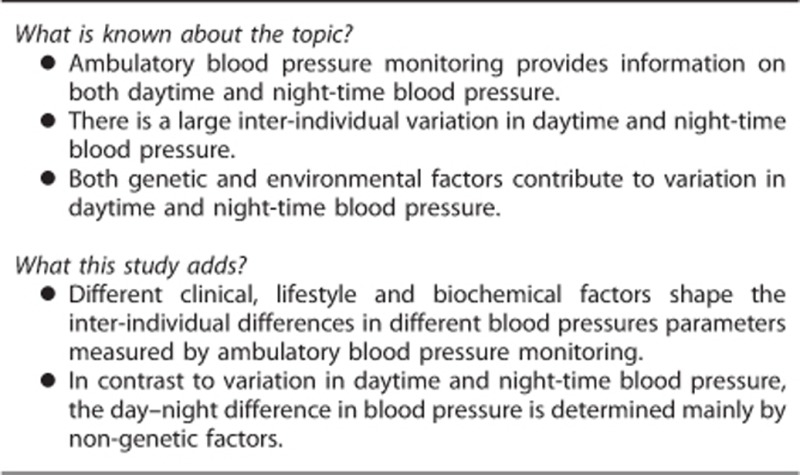


## Figures and Tables

**Table 1:** Demographics of the GRAPHIC study subjects analysed partitioned by generation and sex

*Variable*	*Fathers (377)*	*Mothers (381)*	*Sons (398)*	*Daughters (406)*
Age (years)	53.4 (4.4)	51.6 (4.3)	25.2 (5.0)	25.8 (5.4)
Night SBP (mm Hg)	110.1 (11.0)	104.5 (10.5)	110.4 (8.6)	103.3 (8.0)
Day SBP (mm Hg)	131.2 (12.6)	123.5 (11.9)	127.1 (8.6)	119.1 (8.3)
Night DBP (mm Hg)	66.3 (7.7)	61.7 (7.5)	60.0 (6.5)	58.1 (5.7)
Day DBP (mm Hg)	83.6 (7.9)	77.5 (8.1)	75.2 (7.4)	74.2 (6.1)
SBP percentage decline	13.1 (6.1)	15.2 (6.0)	13.1 (5.8)	13.1 (5.7)
DBP percentage decline	20.6 (7.3)	20.2 (7.2)	19.9 (7.8)	21.5 (7.4)
BMI (kg m^−2^)	27.3 (3.7)	26.5 (4.4)	24.6 (3.9)	24.3 (4.7)
Waist–hip ratio	0.92 (0.07)	0.81 (0.06)	0.86 (0.06)	0.78 (0.07)
Total cholesterol (mmol l^−1^)	5.7 (1.0)	5.9 (1.0)	4.7 (0.9)	4.7 (0.9)
LDL cholesterol (mmol l^−1^)	3.3 (0.7)	3.2 (0.7)	2.5 (0.7)	2.5 (0.6)
HDL cholesterol (mmol l^−1^)	1.3 (0.3)	1.6 (0.4)	1.3 (0.3)	1.5 (0.3)
Triglyceride (mmol l^−1^)	2.0 (1.1)	1.5 (0.8)	1.5 (0.8)	1.2 (0.5)
Serum albumin (g l^−1^)	44.5 (2.5)	44.4 (2.5)	47.0 (2.7)	44.7 (2.8)
Serum urate (μmol l^−1^)	314.1 (63.4)	221 (54.4)	314.8 (63.5)	215.1 (47.8)
Plasma glucose (mmol l^−1^)	5.3 (1.6)	5.1 (1.2)	4.9 (0.8)	4.7 (0.9)
Serum sodium (mmol l^−1^)	139.3 (2.0)	139.3 (2.1)	139.9 (2.0)	138.8 (1.9)
Serum potassium (mmol l^−1^)	4.4 (0.7)	4.3 (0.7)	4.4 (0.6)	4.3 (0.7)
Serum creatinine (μmol l^−1^)	93.6 (11.3)	76.9 (9.2)	89.7 (9.4)	75.7 (9.4)
eGFR ml min^−1^ per 1.73 m^2^	80.7 (11.4)	78.2 (11.1)	103.0 (12.4)	95.4 (13.9)
24-h Urine sodium (mmol)	91.5 (38.4)	65.9 (30.4)	107.4 (49.9)	96.0 (44.2)
24-h Urine potassium (mmol)	50.4 (20.6)	41.0 (15.7)	49.5 (22.0)	48.0 (22.9)
24-h Urine creatinine (mmol)	9.3 (3.9)	5.8 (2.8)	10.6 (5.0)	8.7 (4.2)
CRP mg l^−1^^a^	0.11 (0.10, 0.12)	0.12 (0.11, 0.13)	0.07 (0.06, 0.08)	0.11 (0.10, 0.12)
Alcohol units per week	14.6 (14.8)	7.2 (8.3)	17.4 (15.1)	6.6 (7.5)
Current smoker (%)^b^	51 (13.5)	47 (12.3)	104 (26.1)	99 (24.4)

Abbreviations: BMI, body mass index; CRP, C-reactive protein; DBP, diastolic blood pressure; eGFR, estimated glomerular filtration rate; GRAPHIC Study, Genetic Regulation of Arterial Pressure in Humans in the Community Study; HDL cholesterol, high-density lipoprotein cholesterol; LDL cholesterol, low-density lipoprotein cholesterol; SBP, systolic blood pressure.

Quantitative data are presented as mean (s.d.).

a CRP is presented as geometric mean (confidence interval) due to non-normality.

b Current smoking is presented as number (percentage).

**Table 2:** Multivariable analysis of the association of systolic blood pressure (SBP) phenotypes with other patient characteristics

*Variables*	*Mean day SBP*		*Mean night SBP*		*Day–night decline in SBP*			
	*Beta (s.e.)*	P*-value*	*Beta (s.e.)*	P*-value*	*Beta (s.e.)*	P-*value*	*Beta (s.e.)*^a^	P*-value*^a^
Age	1.418 (0.404)	4.4x10^-4^	−0.281 (0.376)	0.454	1.175 (0.236)	6.5x10^−7^	0.915 (0.225)	4.68 × 10^−5^
Gender^b^	−4.627 (0.762)	1.2x10^−9^	−3.218 (0.709)	5.6x10^−6^	−0.582 (0.445)	0.191	0.277 (0.427)	0.517
Body mass index	1.423 (0.324)	1.2x10 ^−5^	1.034 (0.300)	0.001	0.164 (0.188)	0.383	−0.100 (0.179)	0.578
Total cholesterol	0.772 (0.839)	0.358	−1.287 (0.775)	0.097	1.556 (0.485)	0.001	1.426 (0.459)	0.002
LDL cholesterol	0.673 (0.811)	0.406	1.499 (0.748)	0.045	−0.771 (0.468)	0.099	−0.916 (0.442)	0.038
Triglyceride	0.556 (0.307)	0.071	0.933 (0.285)	0.001	−0.354 (0.178)	0.047	−0.465 (0.169)	0.006
Plasma urate	1.314 (0.388)	0.001	1.149 (0.359)	0.001	−0.043 (0.225)	0.848	−0.287 (0.214)	0.180
Blood glucose	0.29 (0.266)	0.274	0.436 (0.247)	0.078	−0.147 (0.155)	0.343	−0.201 (0.147)	0.172
eGFR	0.69 (0.373)	0.064	0.509 (0.346)	0.14	0.079 (0.216)	0.715	−0.059 (0.205)	0.775
24-h Urine sodium	0.356 (0.287)	0.215	0.478 (0.266)	0.072	−0.113 (0.167)	0.498	−0.174 (0.158)	0.272
C-reactive protein	0.638 (0.261)	0.014	0.514 (0.243)	0.034	0.022 (0.153)	0.888	−0.096 (0.145)	0.506
Alcohol units	1.314 (0.289)	5.3x10^−6^	0.813 (0.268)	0.002	0.221 (0.168)	0.189	−0.015 (0.160)	0.924
Current smoker	−0.357 (0.27)	0.186	−1.302 (0.25)	1.9x10^−7^	0.811 (0.157)	2.2x10^−7^	0.874 (0.148)	3.79 × 10^−9^

Abbreviations: eGFR, estimated glomerular filtration rate; LDL cholesterol, low-density lipoprotein cholesterol.

All beta values are given per standard deviation (s.d.) change in the variable, with a negative beta representing an inverse correlation. Note that the beta values for the day–night decline in BP is a % while those for mean day and night SBP are in mm Hg.

a Additionally adjusted for mean daytime SBP.

b Gender is females compared to males.

**Table 3:** Multivariable analysis of the association of diastolic blood pressure (DBP) phenotypes with other patient characteristics

*Variables*	*Mean day DBP*		*Mean night DBP*		*Day–night decline in DBP*			
	*Beta (s.e.)*	P-*value*	*Beta (s.e.)*	P-*value*	*Beta (s.e.)*	P*-value*	*Beta (s.e.)*^a^	P-*value*^a^
Age	0.161 (0.018)	1.34 × 10^−19^	0.139 (0.017)	8.94 × 10^−17^	-0.005 (0.018)	0.769	−0.838 (0.258)	0.001
Gender^b^	−1.713 (0.61)	0.005	−1.488 (0.574)	0.010	0.393 (0.631)	0.533	0.913 (0.599)	0.127
Waist–hip ratio^b^	11.18 (3.129)	3.53 × 10^−4^	5.611 (2.931)	0.056	3.766 (3.194)	0.238	0.025 (0.26)	0.923
Total cholesterol	1.075 (0.222)	1.31 × 10^−6^	0.299 (0.208)	0.151	0.623 (0.226)	0.006	0.295 (0.239)	0.217
Triglyceride	0.451 (0.256)	0.078	1.081 (0.239)	6.17 × 10^−6^	−0.873 (0.26)	7.87 × 10^−4^	−0.913 (0.215)	2.19 × 10^−5^
Serum albumin	0.02 (0.075)	0.790	−0.094 (0.07)	0.177	0.155 (0.075)	0.039	0.413 (0.202)	0.041
Serum creatinine	−0.032 (0.02)	0.114	0.012 (0.019)	0.528	−0.047 (0.02)	0.022	−0.441 (0.242)	0.069
Plasma urate	0.008 (0.004)	0.030	0.004 (0.003)	0.208	0.003 (0.004)	0.425	0.016 (0.261)	0.950
24-h Urine sodium	0.003 (0.005)	0.508	0.008 (0.004)	0.063	−0.006 (0.005)	0.177	−0.331 (0.199)	0.097
Alcohol units	0.046 (0.016)	0.005	0.014 (0.015)	0.341	0.029 (0.016)	0.076	0.177 (0.201)	0.378
Current smoker	−0.051 (0.497)	0.919	−1.363 (0.465)	0.003	1.676 (0.505)	8.97 × 10^−4^	0.671 (0.189)	3.80 × 10^−4^

All beta values are given per standard deviation (s.d.) change in the variable, with a negative beta representing an inverse correlation. Note that the beta values for the day–night decline in BP is a % while those for mean day and night systolic blood pressure (SBP) are in mm Hg.

a Additionally adjusted for mean daytime SBP.

b Gender is females compared to males.

**Table 4:** Heritability of 24-h, daytime, night-time and day–night differences in SBP and DBP

*Systolic phenotypes*	*24-h SBP*	*Daytime SBP*	*Night-time SBP*	*Day–night SBP decline*^a^
Heritability	0.29 (0.06)	0.18 (0.05)	0.21 (0.06)	0.13 (0.05)^a^
*P*-value	1.00 × 10^−7^	3.44 × 10^−4^	6.70 × 10^−5^	0.006^a^
				
*Diastolic phenotypes*	*24-h DBP*	*Daytime DBP*	*Night-time DBP*	*Day–night DBP decline*^b^
Heritability	0.43 (0.05)	0.39 (0.05)	0.25 (0.06)	0.06 (0.05)^b^
*P*-value	1.33 × 10^−15^	1.27 × 10^−13^	1.80 × 10^−6^	0.119^b^

Abbreviations: DBP, diastolic blood pressure; SBP, systolic blood pressure.

All heritability estimates were adjusted for age, age^2^ and sex.

a Additionally adjusted for mean day SBP.

b Additionally adjusted for mean day DBP.

## References

[bib1] Brotman DJ, Davidson MB, Boumitri M, Vidt DG. Impaired diurnal blood pressure variation and all-cause mortality. Am J Hypertens 2008; 21: 92–97. 18091750 10.1038/ajh.2007.7

[bib2] Fagard RH, Celis H, Thijs L, Staessen JA, Clement DL, De Buyzere ML et al. Daytime and nighttime blood pressure as predictors of death and cause-specific cardiovascular events in hypertension. Hypertension 2008; 51: 55–61. 18039980 10.1161/HYPERTENSIONAHA.107.100727

[bib3] de la Sierra A, Gorostidi M, Banegas JR, Segura J, de la Cruz JJ, Ruilope LM. Nocturnal hypertension or nondipping: which is better associated with the cardiovascular risk profile? Am J Hypertens 2014; 27: 680–687. 24061070 10.1093/ajh/hpt175

[bib4] Hansen TW, Li Y, Boggia J, Thijs L, Richart T, Staessen JA. Predictive role of the nighttime blood pressure. Hypertension 2011; 57: 3–10. 21079049 10.1161/HYPERTENSIONAHA.109.133900

[bib5] Boggia J, Li Y, Thijs L, Hansen TW, Kikuya M, Bjorklund-Bodegard K et al. Prognostic accuracy of day versus night ambulatory blood pressure: a cohort study. Lancet 2007; 370: 1219–1229. 17920917 10.1016/S0140-6736(07)61538-4

[bib6] O'Brien E, Sheridan J, O'Malley K. Dippers and non-dippers. Lancet 1988; 2: 397. 10.1016/s0140-6736(88)92867-x 2899801

[bib7] Hajjar I, Zhao P, Alsop D, Abduljalil A, Selim M, Novak P et al. Association of blood pressure elevation and nocturnal dipping with brain atrophy, perfusion and functional measures in stroke and nonstroke individuals. Am J Hypertens 2010; 23: 17–23. 19798036 10.1038/ajh.2009.187 PMC2810719

[bib8] Ohkubo T, Hozawa A, Yamaguchi J, Kikuya M, Ohmori K, Michimata M et al. Prognostic significance of the nocturnal decline in blood pressure in individuals with and without high 24-h blood pressure: the Ohasama study. J Hypertens 2002; 20: 2183–2189. 12409956 10.1097/00004872-200211000-00017

[bib9] Tobin MD, Raleigh SM, Newhouse S, Braund P, Bodycote C, Ogleby J et al. Association of WNK1 gene polymorphisms and haplotypes with ambulatory blood pressure in the general population. Circulation 2005; 112: 3423–3429. 16301342 10.1161/CIRCULATIONAHA.105.555474

[bib10] Tomaszewski M, Debiec R, Braund PS, Nelson CP, Hardwick R, Christofidou P et al. Genetic architecture of ambulatory blood pressure in the general population: insights from cardiovascular gene-centric array. Hypertension 2010; 56: 1069–1076. 21060006 10.1161/HYPERTENSIONAHA.110.155721 PMC3035934

[bib11] Levey AS, Stevens LA, Schmid CH, Zhang YL, Castro AF 3rd, Feldman HI et al. A new equation to estimate glomerular filtration rate. Ann Intern Med 2009; 150: 604–612. 19414839 10.7326/0003-4819-150-9-200905050-00006 PMC2763564

[bib12] Staessen JA, Bieniaszewski L, O'Brien E, Gosse P, Hayashi H, Imai Y et al. Nocturnal blood pressure fall on ambulatory monitoring in a large international database. The "Ad Hoc' Working Group. Hypertension 1997; 29: 30–39. 9039076 10.1161/01.hyp.29.1.30

[bib13] Almasy L, Blangero J. Multipoint quantitative-trait linkage analysis in general pedigrees. Am J Hum Genet 1998; 62: 1198–1211. 9545414 10.1086/301844 PMC1377101

[bib14] Routledge F, McFetridge-Durdle J. Nondipping blood pressure patterns among individuals with essential hypertension: a review of the literature. Eur J Cardiovasc Nurs 2007; 6: 9–26. 16843730 10.1016/j.ejcnurse.2006.05.001

[bib15] de la Sierra A, Lluch MM, Coca A, Aguilera MT, Sanchez M, Sierra C et al. Assessment of salt sensitivity in essential hypertension by 24-h ambulatory blood pressure monitoring. Am J Hypertens 1995; 8: 970–977. 8845078 10.1016/0895-7061(95)00225-1

[bib16] Verdecchia P, Schillaci G, Guerrieri M, Gatteschi C, Benemio G, Boldrini F et al. Circadian blood pressure changes and left ventricular hypertrophy in essential hypertension. Circulation 1990; 81: 528–536. 2137047 10.1161/01.cir.81.2.528

[bib17] Nishiyama A, Imai Y, Ohkubo T, Tsuji I, Nagai K, Kikuchi N et al. Determinants of circadian blood pressure variation: a community-based study in Ohasama. Tohoku J Exp Med 1997; 183: 1–20. 9453113 10.1620/tjem.183.1

[bib18] Imai Y, Nishiyama A, Ohkubo T, Tsuji I, Nagai K, Kikuchi N et al. Factors affecting the nocturnal decrease in blood pressure: a community-based study in Ohasama. J Hypertens 1997; 15: 827–838. 9280204 10.1097/00004872-199715080-00005

[bib19] Hyman DJ, Ogbonnaya K, Taylor AA, Ho K, Pavlik VN. Ethnic differences in nocturnal blood pressure decline in treated hypertensives. Am J Hypertens 2000; 13: 884–891. 10950396 10.1016/s0895-7061(00)00279-x

[bib20] Kario K, Mitsuhashi T, Shimada K. Neurohumoral characteristics of older hypertensive patients with abnormal nocturnal blood pressure dipping. Am J Hypertens 2002; 15: 531–537. 12074355 10.1016/s0895-7061(02)02266-5

[bib21] Cuspidi C, Macca G, Sampieri L, Fusi V, Severgnini B, Michev I et al. Target organ damage and non-dipping pattern defined by two sessions of ambulatory blood pressure monitoring in recently diagnosed essential hypertensive patients. J Hypertens 2001; 19: 1539–1545. 11564972 10.1097/00004872-200109000-00004

[bib22] Ancoli-Israel S, Stepnowsky C, Dimsdale J, Marler M, Cohen-Zion M, Johnson S. The effect of race and sleep-disordered breathing on nocturnal BP "dipping": analysis in an older population. Chest 2002; 122: 1148–1155. 12377835 10.1378/chest.122.4.1148

[bib23] Omvik P. How smoking affects blood pressure. Blood Press 1996; 5: 71–77. 9162447 10.3109/08037059609062111

[bib24] Minami J, Ishimitsu T, Matsuoka H. Effects of smoking cessation on blood pressure and heart rate variability in habitual smokers. Hypertension 1999; 33: 586–590. 9931170 10.1161/01.hyp.33.1.586

[bib25] Primatesta P, Falaschetti E, Gupta S, Marmot MG, Poulter NR. Association between smoking and blood pressure: evidence from the health survey for England. Hypertension 2001; 37: 187–193. 11230269 10.1161/01.hyp.37.2.187

[bib26] Okubo Y, Miyamoto T, Suwazono Y, Kobayashi E, Nogawa K. An association between smoking habits and blood pressure in normotensive Japanese men. J Hum Hypertens 2002; 16: 91–96. 11850765 10.1038/sj.jhh.1001303

[bib27] Verdecchia P, Schillaci G, Borgioni C, Ciucci A, Zampi I, Battistelli M et al. Cigarette smoking, ambulatory blood pressure and cardiac hypertrophy in essential hypertension. J Hypertens 1995; 13: 1209–1215. 8586813 10.1097/00004872-199510000-00016

[bib28] Mikkelsen KL, Wiinberg N, Hoegholm A, Christensen HR, Bang LE, Nielsen PE et al. Smoking related to 24-h ambulatory blood pressure and heart rate: a study in 352 normotensive Danish subjects. Am J Hypertens 1997; 10: 483–491. 9160757 10.1016/s0895-7061(96)00487-6

[bib29] Hansen KW, Pedersen MM, Christiansen JS, Mogensen CE. Night blood pressure and cigarette smoking: disparate association in healthy subjects and diabetic patients. Blood Press 1994; 3: 381–388. 7704286 10.3109/08037059409102291

[bib30] Schillaci G, Verdecchia P, Borgioni C, Ciucci A, Gattobigio R, Sacchi N et al. Predictors of diurnal blood pressure changes in 2042 subjects with essential hypertension. J Hypertens 1996; 14: 1167–1173. 8906514 10.1097/00004872-199610000-00003

[bib31] Tsioufis C, Syrseloudis D, Dimitriadis K, Thomopoulos C, Tsiachris D, Pavlidis P et al. Disturbed circadian blood pressure rhythm and C-reactive protein in essential hypertension. J Hum Hypertens 2008; 22: 501–508. 18385743 10.1038/jhh.2008.20

[bib32] Draman MS, Dolan E, van der Poel L, Tun TK, McDermott JH, Sreenan S et al. The importance of night-time systolic blood pressure in diabetic patients: Dublin Outcome Study. J Hypertens 2015; 33: 1373–1377. 25882859 10.1097/HJH.0000000000000576

[bib33] Hla KM, Young T, Finn L, Peppard PE, Szklo-Coxe M, Stubbs M. Longitudinal association of sleep-disordered breathing and nondipping of nocturnal blood pressure in the Wisconsin Sleep Cohort Study. Sleep 2008; 31: 795–800. 18548823 10.1093/sleep/31.6.795 PMC2442417

[bib34] Mojon A, Ayala DE, Pineiro L, Otero A, Crespo JJ, Moya A et al. Comparison of ambulatory blood pressure parameters of hypertensive patients with and without chronic kidney disease. Chronobiol Int 2013; 30: 145–158. 23181690 10.3109/07420528.2012.703083

[bib35] Anan F, Takahashi N, Ooie T, Yufu K, Saikawa T, Yoshimatsu H. Role of insulin resistance in nondipper essential hypertensive patients. Hypertens Res 2003; 26: 669–676. 14620920 10.1291/hypres.26.669

[bib36] Okuguchi T, Osanai T, Kamada T, Kimura M, Takahashi K, Okumura K. Significance of sympathetic nervous system in sodium-induced nocturnal hypertension. J Hypertens 1999; 17: 947–957. 10419068 10.1097/00004872-199917070-00011

[bib37] Uzu T, Fujii T, Nishimura M, Kuroda S, Nakamura S, Inenaga T et al. Determinants of circadian blood pressure rhythm in essential hypertension. Am J Hypertens 1999; 12: 35–39. 10075382 10.1016/s0895-7061(98)00182-4

[bib38] International Consortium for Blood Pressure Genome-Wide Association Studies, Ehret GB, Munroe PB, Rice KM, Bochud M, Johnson AD et al. Genetic variants in novel pathways influence blood pressure and cardiovascular disease risk. Nature 2011; 478: 103–109. 21909115 10.1038/nature10405 PMC3340926

[bib39] Wain LV, Verwoert GC, O'Reilly PF, Shi G, Johnson T, Johnson AD et al. Genome-wide association study identifies six new loci influencing pulse pressure and mean arterial pressure. Nat Genet 2011; 43: 1005–1011. 21909110 10.1038/ng.922 PMC3445021

[bib40] Wang X, Ding X, Su S, Yan W, Harshfield G, Treiber F et al. Genetic influences on daytime and night-time blood pressure: similarities and differences. J Hypertens 2009; 27: 2358–2364. 19657283 10.1097/HJH.0b013e328330e84d PMC3713486

[bib41] Fava C, Burri P, Almgren P, Arcaro G, Groop L, Lennart Hulthen U et al. Dipping and variability of blood pressure and heart rate at night are heritable traits. Am J Hypertens 2005; 18: 1402–1407. 16280271 10.1016/j.amjhyper.2005.05.011

[bib42] Kotchen TA, Kotchen JM, Grim CE, George V, Kaldunski ML, Cowley AW et al. Genetic determinants of hypertension: identification of candidate phenotypes. Hypertension 2000; 36: 7–13. 10904005 10.1161/01.hyp.36.1.7

[bib43] Levy D, DeStefano AL, Larson MG, O'Donnell CJ, Lifton RP, Gavras H et al. Evidence for a gene influencing blood pressure on chromosome 17. Genome scan linkage results for longitudinal blood pressure phenotypes in subjects from the framingham heart study. Hypertension 2000; 36: 477–483. 11040222 10.1161/01.hyp.36.4.477

[bib44] Bonati MT, Graziano F, Monti MC, Crocamo C, Terradura-Vagnarelli O, Cirillo M et al. Heritability of blood pressure through latent curve trajectories in families from the Gubbio population study. J Hypertens 2014; 32: 2179–2187. 25275247 10.1097/HJH.0000000000000311

[bib45] Luft FC. Twins in cardiovascular genetic research. Hypertension 2001; 37: 350–356. 11230299 10.1161/01.hyp.37.2.350

[bib46] Parati G, Stergiou G, O'Brien E, Asmar R, Beilin L, Bilo G et al. European Society of Hypertension practice guidelines for ambulatory blood pressure monitoring. J Hypertens 2014; 32: 1359–1366. 24886823 10.1097/HJH.0000000000000221

