# Supplementary material for: Determinants of day–night difference in blood pressure, a comparison with determinants of daytime and night-time blood pressure
Source: J Hum Hypertens. 2016 Mar 17;31(1):43–8. doi: 10.1038/jhh.2016.14 (PMC5144126; doi:10.1038/jhh.2016.14)
Supplement: Supplementary Information [file jhh201614x1.docx]

**Determinants of day-night difference in blood pressure, a comparison with determinants of daytime and night-time blood pressure**

**Muntaser S Musameh et al.**

**Supplementary Table 1:** Univariate analysis of determinants of systolic blood pressure (SBP) phenotypes, after adjustment for age and sex unless otherwise stated

**Supplementary Table 2:** Univariate analysis of the diastolic blood pressure (DBP) phenotypes, after adjustment for age and sex unless otherwise stated.

**Supplementary Figure 1:** Distribution of ratio of night/day difference in systolic (left panel) and diastolic (right panel) BP

**Supplementary Figure 2:** Correlations between two measures of day-night differences in BP - percentage decline (over day time BP) and night-day ratio - for SBP and DBP.

**Supplementary Figure 3:** Correlations between day-night difference in SBP and day-night difference in DBP

**Supplementary Table 1:** Univariate analysis of determinants of systolic blood pressure (SBP) phenotypes, after adjustment for age and sex unless otherwise stated

| Variable | Mean day SBP | | Mean night SBP | | Day-night SBP Percentage Decline | |
| --- | --- | --- | --- | --- | --- | --- |
|  | **Beta (SE)** | **Pvalue** | **Beta (SE)** | **Pvalue** | **Beta† (SE)** | **Pvalue** |
| Age* | 2.377 (0.274) | 4.4x10^-18^ | 0.221 (0.259) | 0.395 | 0.014 (0.001) | 2.3x10^-21^ |
| Sex* | -7.995 (0.523) | 7.5x10^-53^ | -6.399 (0.502) | 3.5x10^-37^ | -0.003 (0.003) | 0.288 |
| Mean day SBP | - | - | 7.0830 (0.249) | 2.9x10^-178^ | 0.020 (0.002) | 2.3x10^-28^ |
| Mean night SBP | 7.365 (0.248) | 2.2x10^-194^ | - | - | -0.030 (0.002) | 3.8x10^-57^ |
| 24 hour SBP | 10.690 (0.114) | <1.0x10^-300^ | 8.840 (0.157) | <1.0x10^-300^ | 0.002 (0.002) | 0.276 |
| Body Mass Index | 2.468 (0.304) | 5.2x10^-16^ | 2.089 (0.269) | 7.9x10^-15^ | 0.000 (0.001) | 0.862 |
| Waist Hip Ratio | 2.060 (0.339) | 1.2x10^-09^ | 1.557 (0.313) | 6.8x10^-07^ | 0.002 (0.002) | 0.426 |
| Total Cholesterol | 1.842 (0.369) | 5.9x10^-07^ | 0.634 (0.331) | 0.056 | 0.007 (0.002) | 4.9x10^-05^ |
| LDL cholesterol | 1.826 (0.365) | 5.5x10^-07^ | 0.854 (0.334) | 0.010 | 0.005 (0.002) | 0.002 |
| HDL cholesterol | -0.505 (0.303) | 0.095 | -0.913 (0.286) | 0.001 | 0.004 (0.002) | 0.024 |
| Triglyceride | 1.716 (0.339) | 4.1x10^-07^ | 1.506 (0.297) | 3.8x^-07^ | 0.000 (0.002) | 0.894 |
| Serum Albumin | 0.390 (0.283) | 0.168 | -0.226 (0.281) | 0.421 | 0.004 (0.002) | 0.007 |
| Serum Urate | 2.498 (0.363) | 5.9x10^-12^ | 2.156 (0.326) | 3.7x10^-11^ | 0.000 (0.002) | 0.836 |
| Blood Glucose | 0.595 (0.263) | 0.024 | 0.729 (0.212) | 5.7x10^-04^ | -0.002 (0.002) | 0.309 |
| Serum Sodium | 0.138 (0.292) | 0.636 | 0.229 (0.267) | 0.391 | 0.000 (0.002) | 0.805 |
| Serum Potassium | -0.302 (0.308) | 0.328 | -0.071 (0.239) | 0.765 | -0.001 (0.001) | 0.295 |
| Serum Creatinine | -0.007 (0.358) | 0.984 | 0.149 (0.321) | 0.641 | -0.002 (0.002) | 0.455 |
| eGFR | 0.103 (0.369) | 0.781 | -0.039 (0.327) | 0.905 | 0.001 (0.002) | 0.509 |
| 24h Urine Sodium | 0.624 (0.268) | 0.020 | 0.865 (0.248) | 4.7x10^-04^ | -0.002 (0.002) | 0.150 |
| 24h Urine Potassium | 0.156 (0.265) | 0.555 | 0.328 (0.263) | 0.212 | -0.002 (0.002) | 0.322 |
| 24h Urine Creatinine | 0.373 (0.29) | 0.199 | 0.570 (0.276) | 0.039 | -0.002 (0.002) | 0.272 |
| C-Reactive Protein | 0.858 (0.399) | 0.031 | 0.803 (0.487) | 0.099 | -0.001 (0.002) | 0.739 |
| Alcohol Units | 1.597 (0.316) | 4.4x10^-07^ | 0.791 (0.262) | 0.002 | 0.004 (0.002) | 0.013 |
| Current Smoker | -0.157 (0.286) | 0.582 | -1.060 (0.25) | 2.3x10^-05^ | 0.008 (0.001) | 1.9x10^-07^ |

**Supplementary Table 2:** Univariate analysis of the diastolic blood pressure (DBP) phenotypes, after adjustment for age and sex unless otherwise stated

| Variable | Mean day DBP | | Mean night DBP | | Day-night DBP Percentage Decline | |
| --- | --- | --- | --- | --- | --- | --- |
|  | **Beta (SE)** | **Pvalue** | **Beta (SE)** | **Pvalue** | **Beta† (SE)** | **Pvalue** |
| Age* | 3.26 (0.175) | 2.6x10^-77^ | 2.657 (0.169) | 1.9x10^-55^ | -0.046 (0.188) | 0.807 |
| Sex* | -3.662 (0.397) | 2.9x10^-20^ | -3.282 (0.367) | 3.5x10^-19^ | 0.627 (0.384) | 0.102 |
| Mean day DBP | - | - | 4.487 (0.183) | 3.8x10^-132^ | 2.674 (0.221) | 1.4x10^-33^ |
| Mean night DBP | 4.678 (0.19) | 4.3x10^-134^ | - | - | -4.744 (0.197) | 4.5x10^-128^ |
| 24 hour DBP | 7.81 (0.082) | <1.0x10^-300^ | 6.139 (0.128) | <1.0x10^-300^ | 0.163 (0.213) | 0.443 |
| Body Mass Index | 0.633 (0.202) | 0.008 | 0.518 (0.189) | 0.006 | -0.020 (0.196) | 0.920 |
| Waist Hip Ratio | 1.418 (0.244) | 5.7x10^-09^ | 0.937 (0.236) | 7.3x10^-05^ | 0.212 (0.264) | 0.422 |
| Total Cholesterol | 1.46 (0.26) | 2.0x10^-08^ | 0.685 (0.227) | 0.003 | 0.550 (0.208) | 0.008 |
| LDL cholesterol | 1.351 (0.254) | 1.0x10^-07^ | 0.666 (0.221) | 0.003 | 0.459 (0.214) | 0.032 |
| HDL cholesterol | -0.056 (0.211) | 0.790 | -0.445 (0.193) | 0.021 | 0.476 (0.202) | 0.019 |
| Triglycerides | 1.11 (0.209) | 1.05x10^-07^ | 1.205 (0.197) | 9.7x10^-10^ | -0.391 (0.212) | 0.064 |
| Serum Albumin | 0.216 (0.199) | 0.278 | -0.197 (0.198) | 0.320 | 0.489 (0.205) | 0.017 |
| Serum Urate | 1.066 (0.25) | 2.0x10^-05^ | 0.857 (0.227) | 1.6x10^-04^ | 0.000 (0.258) | 0.999 |
| Blood Glucose | 0.100 (0.192) | 0.602 | 0.271 (0.212) | 0.201 | -0.237 (0.234) | 0.311 |
| Serum Sodium | -0.017 (0.2) | 0.932 | 0.105 (0.196) | 0.590 | -0.045 (0.199) | 0.820 |
| Serum Potassium | -0.270 (0.206) | 0.190 | -0.049 (0.202) | 0.808 | -0.149 (0.17) | 0.379 |
| Serum Creatinine | -0.099 (0.252) | 0.696 | 0.309 (0.238) | 0.193 | -0.509 (0.264) | 0.054 |
| eGFR | 0.067 (0.259) | 0.797 | -0.272 (0.239) | 0.254 | 0.464 (0.276) | 0.093 |
| 24h Urine Sodium | 0.124 (0.197) | 0.532 | 0.474 (0.174) | 0.007 | -0.425 (0.192) | 0.027 |
| 24h Urine Potassium | 0.088 (0.176) | 0.616 | 0.265 (0.179) | 0.139 | -0.222 (0.193) | 0.250 |
| 24h Urine Creatinine | 0.153 (0.206) | 0.456 | 0.366 (0.198) | 0.065 | -0.256 (0.2) | 0.200 |
| C-Reactive Protein | 0.141 (0.18) | 0.434 | 0.307 (0.282) | 0.277 | -0.233 (0.218) | 0.286 |
| Alcohol Units | 0.871 (0.202) | 1.6x10^-05^ | 0.244 (0.179) | 0.172 | 0.582 (0.227) | 0.010 |
| Current Smoker | 0.106 (0.19) | 0.579 | -0.423 (0.172) | 0.014 | 0.644 (0.187) | 5.6x10^-04^ |

**Legends to Supplementary Tables 1 and 2**

eGFR: estimated glomerular filtration rate; *not adjusted. All beta values are given per standard deviation (SD) change in the variable, with a negative beta representing an inverse correlation. Note that the beta values for the day-night decline in BP is a % while those for mean day and night SBP are in mm Hg.

**Supplementary Figure 1:** Distribution of ratio of night/day difference in systolic (left panel) and diastolic (right panel) BP

**Supplementary Figure 2:** Correlations between two measures of day-night differences in BP - percentage decline (over day time BP) and night-day ratio - for SBP and DBP.

**Supplementary Figure 3:** Correlations between day-night difference in SBP and day-night difference in DBP
